# Plant Microbiomes Alleviate Abiotic Stress-Associated Damage in Crops and Enhance Climate-Resilient Agriculture

**DOI:** 10.3390/plants14121890

**Published:** 2025-06-19

**Authors:** Fazal Ullah, Sajid Ali, Muhammad Siraj, Muhammad Saeed Akhtar, Wajid Zaman

**Affiliations:** 1 College of Life Sciences, Northwest Normal University, Lanzhou 730070, China; fazal@nwnu.edu.cn; 2 Department of Horticulture and Life Science, Yeungnam University, Gyeongsan 38541, Republic of Korea; drsajid@yu.ac.kr; 3 Department of Biotechnology, Jeonbuk National University, Specialized Campus, Iksan 54896, Republic of Korea; sirajuom2@gmail.com; 4 Department of Chemistry, Yeungnam University, Gyeongsan 38541, Republic of Korea; 5 Department of Life Sciences, Yeungnam University, Gyeongsan 38541, Republic of Korea

**Keywords:** plant microbiomes, abiotic stress, climate change, crop plants, microbial consortia, synthetic biology, stress resilience, metagenomics, sustainable agriculture

## Abstract

Plant microbiomes, composed of a diverse array of microorganisms such as bacteria, fungi, archaea, and microalgae, are critical to plant health and resilience, playing key roles in nutrient cycling, stress mitigation, and disease resistance. Climate change is expected to intensify various abiotic stressors, such as drought, salinity, temperature extremes, nutrient deficiencies, and heavy metal toxicity. Plant-associated microbiomes have emerged as a promising natural solution to help mitigate these stresses and enhance agricultural resilience. However, translating laboratory findings into real-world agricultural benefits remains a significant challenge due to the complexity of plant–microbe interactions under field conditions. We explore the roles of plant microbiomes in combating abiotic stress and discuss advances in microbiome engineering strategies, including synthetic biology, microbial consortia design, metagenomics, and CRISPR-Cas, with a focus on enhancing their practical application in agriculture. Integrating microbiome-based solutions into climate-smart agricultural practices may contribute to long-term sustainability. Finally, we underscore the importance of interdisciplinary collaboration in overcoming existing challenges. Microbiome-based solutions hold promise for improving global food security and promoting sustainable agricultural practices in the face of climate change.

## 1. Introduction

Crop productivity is projected to increase by more than 60 percent by 2050 to sustain the growing global population, which is expected to reach 9.7 billion. The United Nations forecasts that the global population will surge to 10.3 billion by 2084, an increase of 2.1 billion from 2024, emphasizing the pressing challenge of meeting the mounting demand for food and other vital resources to support this unprecedented population growth [1,2]. However, achieving this target is hindered by the dual challenges of climate change and the limitations of current agricultural practices. A significant barrier lies in the utilization of arid and degraded land, where enhancing crop yields remains particularly challenging [3]. In an attempt to improve agricultural output, some farmers rely on synthetic chemical fertilizers, which, while increasing short-term yields, pose long-term risks to soil health, biodiversity, and the integrity of the food chain [4]. Additionally, the high economic costs associated with chemical fertilizers, coupled with their negative impacts on plant physiology, further complicate sustainable agricultural practices. While some microorganisms are recognized as harmful to plants due to their pathogenic properties, the majority of soil microorganisms play a crucial role in supporting plant survival under stressful conditions [5]. These beneficial microorganisms are increasingly utilized in agriculture to enhance crop production. They contribute significantly to various processes, including the biological fixation of atmospheric nitrogen, decomposition of organic matter, detoxification of pesticides, suppression of plant diseases, and the production of various bioactive compounds, plant hormones, and enzymes [6,7].

Plant microbiomes, composed of diverse microorganisms such as bacteria, fungi, archaea, and microalgae, are essential for enhancing plant resilience, particularly under abiotic stresses like drought and salinity [8,9]. These microorganisms inhabit distinct plant regions—such as the rhizosphere, phyllosphere, and endosphere—and play vital roles in supporting plant health. In the rhizosphere, plants provide carbon sources, such as sugars and amino acids, to microorganisms through root exudates, which support microbial growth [10]. In other words, the rhizosphere is the near vicinity of plant roots, which plays an important role in the nutrient cycling of crops and soil organic nitrogen mineralization [11,12]. In return, these microbes improve plant health by promoting nutrient acquisition, enhancing root growth, and protecting against pathogens [13]. For example, *Azospirillum* spp. in the rhizosphere improves plant water-use efficiency and nutrient uptake during drought conditions by producing osmolytes like proline and trehalose [14]. Similarly, microbes in the phyllosphere help protect plants from pathogens and mitigate environmental stress by modulating plant hormones like abscisic acid (ABA) and auxins. The endosphere, which hosts microbes within plant tissues, also contributes to plant health by regulating hormone levels, enhancing nutrient uptake, and promoting overall plant vitality [15]. Together, these microbial communities form a mutualistic relationship with plants, helping them resist and recover from abiotic stresses, thereby improving crop resilience and productivity [16]. The microbial communities are pivotal for ecosystem resilience, contributing to both resistance and resilience, especially in the face of climate change [17]. Plant–microbiome interactions also play a significant role in enhancing ecosystem resilience by supporting plant health and restoring soil vitality [18]. Additionally, plant-associated microbiomes act as natural mediators of plant stress responses, enabling plants to recover from environmental disturbances. By fostering plant health, microbiomes help mitigate the effects of climate change on ecosystems and promote the restoration of ecological functions, such as nutrient cycling and carbon sequestration [19]. Microbiomes also reduce the need for chemical fertilizers and pesticides by improving plant resilience to environmental stressors [20].

However, the intensification of abiotic stress factors due to climate change presents a growing challenge to global food production. Rising temperatures, prolonged droughts, salinity, and extreme weather events threaten to reduce crop yields and exacerbate food insecurity [21]. It is projected that climate change could cause a 20–30% reduction in global crop yields by 2050, with the most significant losses occurring in regions already facing water scarcity and extreme temperatures [22]. These abiotic stresses hinder plant physiological processes, such as photosynthesis, nutrient uptake, and water regulation, leading to reduced crop productivity and quality. As the unpredictability of climate patterns increases, the ability of plants to withstand these stressors becomes increasingly important [23]. On the other hand, plant microbiomes offer a promising solution to mitigate the effects of abiotic stress, providing a natural and sustainable means to enhance plant resilience and safeguard food security in a rapidly changing climate [24]. Recent advancements in microbiome research have led to the development of new strategies for leveraging plant–microbiome interactions in agriculture. Innovative tools such as the application of biofertilizers, microbial plant biostimulants, and synthetic microbial communities (SynComs) represent promising strategies to support plant health and resilience under stress conditions [25]. Biofertilizers containing beneficial microorganisms—such as nitrogen-fixing bacteria, phosphorus-solubilizing fungi, and microalgae—improve nutrient availability and soil fertility, thereby enhancing plant growth [26]. Similarly, microbial biostimulants are designed to enhance plant growth and resilience, particularly under stressful conditions. These biostimulants work by stimulating beneficial microbial activity on plant surfaces and in the rhizosphere [27]. SynComs, which involve the design of microbial communities tailored to specific plant needs, are an emerging approach with the potential to improve plant stress tolerance. By combining different microbial species, SynComs can optimize nutrient acquisition, improve resistance to drought or salinity, and promote overall plant health. SynComs in the rhizosphere are primarily influenced by root exudates, a complex array of organic compounds secreted by plant roots. These exudates act as chemical cues, selectively attracting and recruiting beneficial microorganisms. This dynamic interaction plays a crucial role in enhancing plant–microbe symbiosis, which in turn contributes to improved plant health, resilience, and productivity [28,29]. These microbiome-based solutions provide a sustainable alternative to chemical fertilizers and pesticides, helping to mitigate the negative environmental impacts of conventional agricultural practices [30].

The integration of microbiomes into agricultural practices aligns with the United Nations’ Sustainable Development Goal (SDG) 12 on responsible consumption and production. SDG 12 emphasizes the need to promote sustainable agricultural practices, reduce reliance on chemical fertilizers and pesticides, and support soil health management [31,32]. Microbial-based technologies, such as bioinoculants and microbial biostimulants, offer a means to reduce the environmental footprint of farming by improving nutrient cycling, enhancing soil fertility, and promoting biodiversity. These solutions not only reduce dependence on harmful agrochemicals but also contribute to the restoration and preservation of soil ecosystems [33]. By improving soil health, increasing plant resilience, and supporting sustainable farming practices, microbiomes offer a pathway to more sustainable and climate-resilient agricultural systems [34].

The aim of this review is to explore the multifaceted roles of plant microbiomes in mitigating abiotic stresses, particularly in the context of climate change. We examine the latest research on plant–microbiome interactions, focusing on how these microbiomes contribute to plant stress resilience. The review also discusses the potential applications of biofertilizers, microbial biostimulants, and SynComs for enhancing plant resilience to climate-related stressors. Furthermore, we explore the interactions between different microbial communities within the plant microbiome and their implications for stress mitigation. The review also highlights the relevance of these findings to global sustainability goals, particularly in relation to sustainable developmental goal (SDG 12). Finally, we aim to identify gaps in our current knowledge and suggest directions for future research that could help bridge these gaps, fostering the development of microbiome-based solutions for climate-resilient and sustainable agriculture.

## 2. Abiotic Stress in Plants: Impacts and Resilience

Abiotic stress factors can occur naturally, but their frequency and intensity are increasing due to climate change. Abiotic stresses, intensified by climate change, significantly impact plant growth and crop production, particularly in low-latitude regions of developing countries. Rising carbon dioxide levels and temperatures threaten agricultural yields, intensifying competition for resources [24,35]. This underscores the need for developing climate-resilient crops to ensure food security and environmental sustainability. Abiotic stresses like drought, salinity, and temperature extremes disrupt key plant processes such as photosynthesis, transpiration, and nutrient uptake, leading to reduced plant growth and productivity. For example, drought leads to water scarcity, salinity causes osmotic stress, and extreme temperatures (hot or cold) can damage proteins and membranes. These stresses not only directly affect plant health but also reduce biodiversity and ecosystem services, thus impacting agricultural productivity and sustainability [36,37]. Abiotic stresses interfere with critical plant processes, including photosynthesis, transpiration, and nutrient uptake. For example, salinity stress causes osmotic imbalance, affecting cellular functions and root growth. Extreme temperatures, whether heat or cold, can damage cellular structures, proteins, and membranes, ultimately impairing plant development and reducing productivity. As a result, crops under abiotic stress often exhibit stunted growth, reduced reproductive success, and lower resistance to diseases, making them more vulnerable to environmental changes, especially in the context of climate change. An overview of these major abiotic stresses, along with their typical physiological disruptions, is summarized in Table 1. 

However, plant-associated microbes play a crucial role in mitigating the adverse effects of abiotic stresses on plants. These microorganisms can enhance plant resilience through various mechanisms, such as improving nutrient uptake, promoting water retention, and producing growth-promoting hormones. The manipulation of microbial communities in the soil or through inoculation strategies offers promising avenues for developing climate-resilient crops capable of withstanding the growing challenges posed by abiotic stresses.

### 2.1. Drought Stress

**Figure 1:**
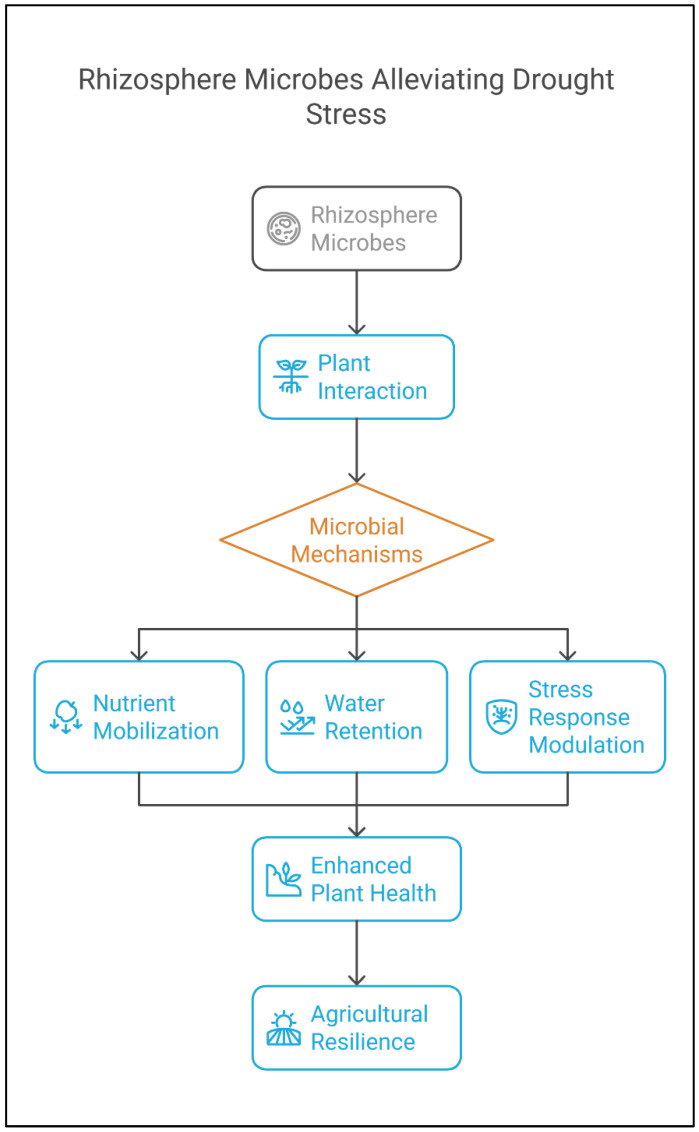
Flowchart illustrating the rhizosphere microbes alleviate drought stress.

Drought is a primary abiotic stress affecting plant growth worldwide. It limits water availability, causing physiological disruptions such as stomatal closure, reduced photosynthesis, and impaired nutrient uptake [44]. Drought stress triggers a complex array of plant responses, affecting multiple physiological, biochemical, and molecular processes that ultimately influence plant growth and productivity [45]. These responses range from alterations in cellular metabolism to significant changes in growth rates and crop yields (Figure 1). Understanding the mechanisms behind drought-induced changes is crucial for developing strategies to improve plant resistance to water-limited environments. Under drought conditions, plants exhibit a range of morphological and physiological changes, including reduced stomatal conductance, which limits CO_2_ assimilation and disrupts photosynthesis. As a result, drought stress leads to a decrease in leaf size, stunted stem elongation, and reduced root proliferation, which collectively impair the plant’s ability to absorb water and nutrients. The reduction in water-use efficiency further exacerbates these effects, leading to a decline in overall plant growth and yield [46]. 

At the biochemical level, drought stress triggers the accumulation of osmolytes, such as proline, sugars, and betaine, which play critical roles in protecting plant cells from dehydration and maintaining cellular function under water stress. Additionally, drought stress induces the generation of reactive oxygen species (ROS), which can cause oxidative damage to cellular components such as lipids, proteins, and nucleic acids. Plants have evolved sophisticated antioxidative systems to control ROS production and mitigate their harmful effects, ensuring cellular integrity during prolonged drought exposure. These systems, including enzymes like superoxide dismutase (SOD), catalase (CAT), and peroxidases, function in concert to scavenge ROS and maintain cellular homeostasis [47]. Understanding the intricate interplay of these physiological and biochemical responses is essential for advancing drought tolerance research and improving crop resilience to water scarcity. Microbial communities, particularly those in the rhizosphere, play a critical role in enhancing plant tolerance to drought by improving nutrient acquisition, enhancing root growth, and producing phytohormones that augment plant resilience. Microorganisms improve water-use efficiency by promoting root growth and producing osmolytes like proline and trehalose [48]. Similarly, Ansari et al. [49] reported a drought-tolerant strain of *Pseudomonas azotoformans* (FAP5), which produces exopolysaccharides, indole 3 acetic acid, and solubilized tricalcium phosphate and exhibits ACC deaminase activity. The inoculation of the FAP5 strain on wheat plants showed significant improvement in growth attributes, photosynthetic pigment efficiency, and other physiological attributes under drought-stress conditions. Similarly, significant antioxidative enzymatic activities in FAP5-inoculated plants were observed compared to uninoculated plants. 

Moreover, PGPR’s role in enhancing plant drought resistance is closely linked to both plant species and the intensity of drought stress. C4 plants, which utilize the C4 photosynthetic pathway, exhibit greater adaptation to arid conditions compared to C3 plants. Studies have shown that the contribution of the beneficial microbiome to drought resistance varies between C3 and C4 plants. Under drought stress, the efficient photosynthetic assimilation pathway in C4 plants facilitates the regulation of their antioxidant systems, leading to the secretion of greater amounts of organic acids. This enhances the formation of a more compact rhizosphere soil microenvironment compared to C3 plants [50]. Additionally, these microbes influence plant hormone levels, particularly abscisic acid (ABA), to regulate stomatal closure and conserve water under drought conditions. Research into the microbial mechanisms of drought tolerance has advanced significantly, highlighting their potential for agricultural applications to combat water scarcity [51].

### 2.2. Salinity Stress

Salinity stress is a major threat to plant growth, particularly in regions with high soil salinity. High salt concentrations reduce the osmotic potential of the soil, which limits water uptake and leads to dehydration [52]. Salinity stress triggers various biochemical and molecular changes in plants, primarily through the production of reactive oxygen species (ROS), which can lead to significant anatomical and physiological alterations. Excessive ROS accumulation is highly detrimental to plants, causing cellular damage such as chlorophyll degradation, cell membrane oxidation, and ultimately, cell death. To counteract the harmful effects of elevated ROS levels, plants activate antioxidant defense mechanisms [53,54]. Well-known antioxidant enzymes, such as superoxide dismutase (SOD) and reduced glutathione (GSH), play a crucial role in neutralizing the free radicals generated during salinity stress. Studies have demonstrated a significant enhancement of the antioxidant defense system, which contributes to increased adaptability and survival of plants in stressful conditions. High concentrations of salt (NaCl) induce ionic imbalance, leading to osmotic stress and ionic toxicity. Sodium (Na^+^) and potassium (K^+^) ions are essential in plant physiology under salt stress, with the expulsion of Na^+^ and the influx of K^+^ being critical strategies to mitigate salinity stress. The imbalance caused by excessive Na^+^ influx disrupts potassium uptake by roots, resulting in water deficiency and osmotic stress, which in turn negatively affects plant growth [55]. This can manifest as halted photosynthesis, reduced stomatal conductance, abnormal transpiration rates, and lower chlorophyll concentrations. 

Microbial communities, including halophilic microbes such as *Halomonas* and *Arthrobacter*, help mitigate salinity stress by producing osmolytes like glycerol and betaine, which help plants maintain cellular hydration and structural integrity. These microbes also enhance ion regulation, assisting in the exclusion or compartmentalization of toxic ions like Na^+^, which reduces ion toxicity and prevents cellular damage [56]. Recent research has identified halotolerant plant growth-promoting rhizobacteria (PGPR), such as *Acinetobacter*, *Azotobacter*, *Bacillus* sp., *Serratia* sp., *Pseudomonas* sp., and *Rhizobium* sp., which enhance plant growth under both normal and saline conditions. These bacteria contribute to plant health by nitrogen fixation, solubilizing inorganic phosphate, and producing siderophores and phytohormones. Additionally, various studies have highlighted the positive impact of halotolerant microbes on the growth and development of several crops, including rice, wheat, maize, tomato, soybean, lettuce, cotton, pepper, and canola, under salinity stress [53,57]. Furthermore, plant growth-promoting rhizobacteria (PGPR), such as *Bacillus* species, facilitate salt tolerance through mechanisms like Na^+^-ATPase pumps, which help exclude excess sodium from plant tissues. These microbial strategies play a crucial role in helping plants maintain growth and productivity under saline conditions [58].

### 2.3. Temperature Extremes (Heat and Cold Stress)

Extreme temperature significantly affects plant physiology and growth. Heat stress leads to protein denaturation, membrane disruption, and inhibited enzymatic activity, while cold stress can cause intracellular ice formation and metabolic disruptions [59,60]. Cold and heat stress are both significant environmental challenges that affect plant growth and yield, with rice being particularly vulnerable to both extremes. Cold stress during late spring can lead to the accumulation of ROS, causing damage to cellular structures such as membranes, proteins, and nucleic acids. This disrupts photosynthesis, water absorption, and overall plant function. Heat stress, on the other hand, causes protein denaturation, enzyme inactivation, and increased ROS production, which can lead to similar cellular damage and reduced crop yield [61]. Both types of stress induce oxidative damage, electrolyte leakage, and cellular ion imbalances, severely impacting plant growth and development. While traditional methods to mitigate cold and heat stress, such as artificial warming, plastic coverings, and irrigation, are costly and environmentally problematic, plant growth-promoting rhizobacteria (PGPR) offer a more sustainable solution [62]. 

PGPR colonizes the plant rhizosphere and enhances temperature stress tolerance. These microbes help mitigate stress effects by producing phytohormones, improving nutrient availability, reducing oxidative stress, and regulating gene expression related to stress tolerance. Specifically, PGPR can help plants by enhancing photosynthesis, reducing osmotic stress, scavenging excess ROS, and improving nutrient uptake, which all contribute to better stress resilience [63]. Studies have shown that microbial communities from stress-resistant plants, such as peas, can be enriched and applied to improve stress tolerance in crops like rice. These microbial communities, especially those containing nitrogen-fixing bacteria, not only enhance cold and heat stress tolerance but also improve soil quality and promote plant growth. By supporting the growth of beneficial microbes in the rhizosphere, PGPR can act as an effective and eco-friendly approach to help plants withstand both cold and heat stress, ultimately improving agricultural productivity under adverse environmental conditions [64]. Microbial communities in the rhizosphere help mitigate temperature extremes by producing heat shock proteins (HSPs) and cold shock proteins (CSPs), which stabilize cellular structures and enzymes [65]. Notably, psychrotrophic and psychrophilic soil bacteria, such as Pseudomonas putida and Sinorhizobium meliloti, have been shown to synthesize CSPs that enhance cold stress tolerance by stabilizing mRNA and facilitating ribosome function under low-temperature conditions [65,66]. Certain microbes, like *Bacillus subtilis* and *Trichoderma* spp., also enhance plant tolerance to temperature stress by producing compatible solutes like trehalose and glycerol, which protect cellular membranes from heat-induced denaturation and cold-induced crystallization [67].

### 2.4. Nutrient Deficiencies

Inadequate availability of essential nutrient elements in the soil is one of the primary challenges to plant growth in agricultural areas, mostly in tropical regions. Tropical soils often suffer from low nutrient content due to high rates of weathering and leaching. Plant growth-promoting rhizobacteria (PGPR) play a crucial role in enhancing soil nutrient availability through various mechanisms. These bacteria actively participate in the geochemical cycles of nutrients and influence the bioavailability of essential elements to plants [68]. PGPR promotes plant growth by enhancing nutrient uptake, improving soil structure, and facilitating nutrient cycling. The use of PGPR as bio-inoculants not only boosts the availability of vital nutrients but also reduces the reliance on chemical fertilizers, minimizing environmental pollution and supporting more sustainable agricultural practices [69]. PGPR contributes to nutrient availability through several key mechanisms, including nitrogen fixation, phosphorus solubilization, potassium mobilization, and the production of siderophores that chelate micronutrients, making them more accessible to plants. These processes help to increase the bioavailability of macronutrients such as nitrogen (N), phosphorus (P), potassium (K), and sulfur (S), as well as micronutrients like iron (Fe) and manganese (Mn) [68,70]. By promoting the cycling of these essential elements, PGPR enhances plant nutrition and growth, especially in nutrient-deficient soils. This microbial action not only contributes to improving soil fertility but also offers an eco-friendly alternative to chemical fertilizers, aligning with sustainable agricultural practices aimed at reducing environmental impact and improving crop yields.

Nutrient deficiencies, particularly of nitrogen, phosphorus, and potassium, can have a debilitating effect on plant growth and productivity. These macronutrients are essential for key metabolic processes, including protein synthesis, energy transfer, and cellular structure formation [71]. Nitrogen is a major component of amino acids and chlorophyll, and its deficiency leads to poor protein synthesis, stunted growth, and chlorosis. Phosphorus is critical for energy transfer and root development, and its deficiency impairs root function, reduces flower and fruit production, and limits overall plant growth [72,73]. Potassium is involved in regulating water balance, enzyme activation, and stress tolerance, and its deficiency compromises the plant’s ability to withstand other stressors, such as drought and heat [74]. In many agricultural systems, nutrient deficiencies arise due to poor soil fertility, imbalanced fertilizer application, or disruptions in nutrient cycling [75]. These deficiencies reduce crop yields and quality, particularly in regions where the availability of fertilizers is limited or where soil degradation is widespread. Furthermore, nutrient deficiencies often interact with other abiotic stresses, exacerbating their effects on plant health and productivity [76,77].

## 3. Plant Microbiomes and Stress Mitigation

**Figure 2:**
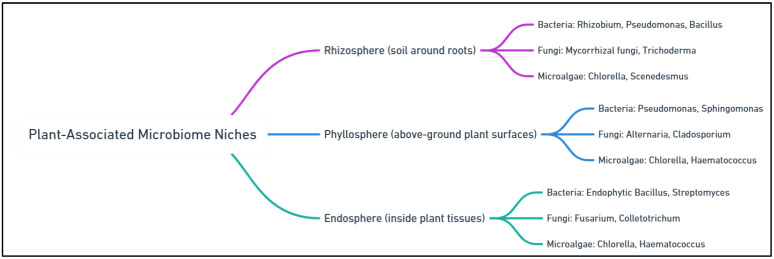
Plant-associated microbiome: key niches and microorganisms.

The plant microbiome plays a crucial role in protecting the host against abiotic stress by enhancing the plant’s ability to acquire nutrients and develop tolerance to environmental challenges. The metabolic potential of the microbiome is vast, significantly supplementing the plant’s own metabolic capacity [78]. The plant microbiome is highly dynamic, with its composition and structure shaped by the intensity and duration of abiotic stress. In response to such stress, plants and their associated microbiomes work synergistically, forming a mutualistic relationship that supports both survival and growth under challenging conditions [79]. The interaction between plants and their microbiomes is fundamentally mutualistic. While plants provide nutrients and habitat for microorganisms, such as carbon sources in root exudates, microorganisms support plant health by enhancing nutrient uptake, promoting growth, and helping mitigate stress [80,81]. For example, *Azospirillum* spp. in the rhizosphere helps promote root growth, enhancing nutrient uptake, while *Bacillus* spp. produce antifungal compounds that protect plants from harmful pathogens. Moreover, researchers have highlighted the significant role of soil microbial flora in enhancing plant growth under challenging environmental conditions. Specifically, microbes from genera such as *Pseudomonas, Variovorax*, *Bacillus*, *Azotobacter*, *Enterobacter*, *Aeromonas*, and *Klebsiella* have been shown to positively influence plant growth and resilience. These microbial species contribute to plant health through various mechanisms, including nitrogen fixation, nutrient solubilization, production of phytohormones, and suppression of harmful pathogens. For instance, *Azospirillum* and *Azotobacter* are well-known for their ability to fix atmospheric nitrogen, enriching the soil and making this essential nutrient available to plants. Similarly, species like Bacillus and Pseudomonas produce plant growth-promoting substances, such as indole-3-acetic acid (IAA), which help in root development and overall plant growth [82]. In addition to these beneficial interactions, the microbial communities also enhance the plant’s tolerance to abiotic stresses such as drought, salinity, and temperature extremes. Certain bacteria, such as *Klebsiella* and *Enterobacter*, are involved in the synthesis of osmoprotectants that help plants mitigate the adverse effects of water stress. Moreover, these microbes can stimulate the plant’s defense mechanisms by triggering systemic acquired resistance (SAR), which improves the plant’s ability to cope with pathogens. The presence of these microorganisms not only promotes growth but also boosts the plant’s overall health and survival in adverse environments, thus offering a sustainable approach to improving agricultural productivity, especially in regions facing environmental stresses [83]. Additionally, microbes in the rhizosphere can stimulate the plant’s immune system, enabling it to better withstand abiotic stresses like drought and salinity. This bidirectional exchange strengthens the plant’s resilience against environmental challenges, fostering plant survival and productivity under adverse conditions [84]. Figure 2 illustrates different plant-associated microbial niches and highlights some representative organisms within each.

### 3.1. Composition and Specific Microbial Groups in Plant Microbiomes

The composition of plant microbiomes varies significantly based on environmental conditions, plant species, developmental stages, and agricultural practices. Recent metagenomic analyses have revealed specific microbial groups consistently associated with enhanced plant resilience. For example, rhizosphere microbiomes often contain beneficial species such as *Azospirillum brasilense*, *Bacillus subtilis*, *Pseudomonas fluorescens*, and arbuscular mycorrhizal fungi (*Glomus intraradices*), which improve nutrient acquisition and stress tolerance [85,86]. Endophytic communities frequently include bacteria such as *Enterobacter cloacae* and *Burkholderia cepacia*, as well as fungal endophytes like *Trichoderma harzianum*, known for their roles in promoting plant growth and pathogen resistance [87]. Phyllosphere communities commonly harbor stress-adaptive species, including *Methylobacterium extorquens* and *Sphingomonas melonis*, contributing to plant resilience against environmental stressors [88,89]. Additionally, plant growth-promoting microalgae (PGPMAs) such as *Chlorella vulgaris* and *Spirulina platensis* have recently gained attention due to their beneficial roles in nutrient provision and abiotic stress alleviation [90]. Understanding these specific microbial compositions can guide targeted microbiome management practices to enhance agricultural productivity under abiotic stress conditions. 

### 3.2. Mechanisms of Stress Alleviation

Plant microbiomes alleviate stress through a range of mechanisms that enhance plant resilience [91]. These include enhanced nutrient uptake, the regulation of plant hormonal pathways, the production of stress-relieving metabolites, and the induction of systemic resistance [8]. Table 2 summarizes the principal microbial mechanisms that alleviate abiotic stress in plants. Understanding these mechanisms is essential for guiding microbiome optimization strategies—for instance, by selecting or engineering microbial strains with specific functional traits that can be harnessed through bioinoculants, synthetic consortia, or plant-microbe matching for improved crop stress tolerance under field conditions.

#### 3.2.1. Enhanced Nutrient Uptake

One of the primary roles of plant-associated microbes is to enhance nutrient uptake, particularly under stress conditions where nutrient availability may be limited. In the rhizosphere, certain bacteria and fungi enhance the plant’s access to vital nutrients such as nitrogen, phosphorus, and potassium [98]. For instance, free-living diazotrophs such as *Azotobacter vinelandii* are capable of fixing atmospheric nitrogen in the soil without plant association, contributing to soil fertility, particularly in nutrient-poor environments [99]. In symbiotic associations, genera like *Rhizobium*, *Bradyrhizobium*, *Mesorhizobium*, and *Sinorhizobium* form highly specialized mutualistic relationships with leguminous plants (Fabaceae), colonizing root or stem nodules where nitrogen fixation occurs in a controlled microaerobic environment [100]. These interactions are highly specific and involve complex signaling between plant roots and rhizobia, ensuring effective colonization and nitrogen assimilation. Additionally, actinobacteria such as *Frankia* form similar symbiotic associations with actinorhizal plants, contributing significantly to nitrogen cycling in forests and marginal ecosystems [101]. Fungal species like *Penicillium* and *Aspergillus* also contribute to nutrient acquisition by solubilizing phosphorus, thus improving availability in otherwise inaccessible forms [102]. In saline or nutrient-poor soils, these microbes play a vital role in alleviating the negative effects of nutrient deficiencies and can enhance the uptake of essential micronutrients such as iron and magnesium by producing siderophores that chelate and transport these ions to plant roots [103].

#### 3.2.2. Regulation of Plant Hormonal Pathways

Plant microbiomes also regulate plant stress responses by modulating plant hormonal pathways. Key hormones, such as ABA, auxins, and cytokinins, play pivotal roles in mediating plant responses to stress [104]. Abscisic acid, for example, is a hormone that regulates stomatal closure during drought, which helps conserve water. Microorganisms in the rhizosphere and endosphere can influence the synthesis and action of ABA, thus enhancing plant water-use efficiency during periods of water scarcity [105]. Similarly, auxins and cytokinins, involved in cell division and growth, can be modulated by microbial communities to regulate root architecture and stimulate growth under stress, as recently demonstrated by auxin production in the microalga Chlamydomonas [106]. In addition, specific microbial strains such as *Azospirillum brasilense*, *Pseudomonas fluorescens*, and *Bacillus subtilis* can directly synthesize phytohormones like auxins and cytokinins, enhancing plant growth and stress tolerance through improved root and shoot development [107].

#### 3.2.3. Production of Stress-Relieving Metabolites

Microbes within the plant microbiome produce a variety of secondary metabolites that help alleviate plant stress. These stress-relieving metabolites include osmolytes, antioxidants, and other protective compounds that enhance the plant’s ability to cope with environmental stressors [108]. Osmolytes, such as proline and trehalose, help plants maintain cell turgor and protect cellular structures during periods of dehydration or osmotic stress [109]. Antioxidants, such as polyphenols and flavonoids, counteract the damaging effects of ROS, which accumulate during oxidative stress and can lead to cellular damage. By scavenging ROS, these antioxidants help protect plant cells from oxidative damage caused by high temperatures, drought, and other environmental stressors [110]. Microbial metabolites, such as volatile organic compounds (VOCs) produced by Bacillus subtilis and lipopeptides like surfactin, induce protective enzymes including superoxide dismutase (SOD), catalase (CAT), and peroxidases (POD), thereby strengthening plant defense mechanisms and enhancing abiotic stress tolerance [111].

#### 3.2.4. Induction of Systemic Tolerance and Improved Root Architecture 

In addition to local stress mitigation, plant microbiomes can induce systemic tolerance across the entire plant. This is achieved through signaling pathways that enable the plant to activate stress tolerance mechanisms in distant tissues, even in areas not directly exposed to the stressor [92]. For instance, beneficial microbes such as *Pseudomonas fluorescens*, *Bacillus amyloliquefaciens*, and *Trichoderma harzianum* in the rhizosphere can trigger systemic acquired resistance pathways, enhancing the plant’s ability to respond effectively to subsequent abiotic stresses [112]. Additionally, the presence of microbes can improve root architecture, better equipping the plant to absorb water and nutrients under stressful conditions [113]. Microbes can influence root growth patterns by promoting the formation of lateral roots and root hairs, increasing the surface area for nutrient uptake. The enhanced root architecture is particularly important under drought and salinity stress, when plants need to access deeper soil layers to obtain water and nutrients [114].

In addition to their direct effects on the plant, microbiomes can enhance soil health, improving its structure and fertility, which further supports plant growth under stress conditions [115]. By improving nutrient availability and supporting healthy root systems, the plant microbiome plays a crucial role in building resilience against a wide range of environmental challenges [116].

#### 3.2.5. Heavy Metal Detoxification

Plant microbiomes also contribute to abiotic stress mitigation by detoxifying heavy metals in contaminated soils. Microorganisms such as *Pseudomonas putida*, *Bacillus subtilis*, and *Azotobacter chroococcum* can sequester toxic metals like cadmium, lead, and arsenic through mechanisms such as biosorption, bioaccumulation, and precipitation. These microbes produce metal-binding proteins, exopolysaccharides, and chelating agents like siderophores, which bind heavy metals and reduce their bioavailability to plants [117,118]. Rhizobia, such as *Rhizobium leguminosarum*, are also known to synthesize large amounts of EPS that help protect plants from drought, cold stress, and metal toxicity, particularly cadmium and zinc, during symbiosis with legumes [119,120]. Additionally, sulfate-reducing bacteria such as *Desulfovibrio desulfuricans* can transform toxic metal ions into less harmful forms through biotransformation. These microbial strategies not only enhance plant survival under metal stress but also contribute to phytoremediation and improved soil health [121]. The main microbial mechanisms for metal stress management differ in how metals are processed. Biosorption refers to the passive adsorption of metal ions to microbial cell walls without metabolic activity [122]. Bioaccumulation involves active, energy-dependent uptake and internal storage of heavy metals by microbial cells. Biotransformation, in contrast, relies on enzymatic activity to chemically modify toxic metals into less harmful or inert forms, often changing their oxidation state [123]. Table 3 provides examples of microbial strategies for mitigating heavy metal stress in various contexts.

## 4. Impacts of Climate Change on Plant–Microbe Interactions

**Figure 3:**
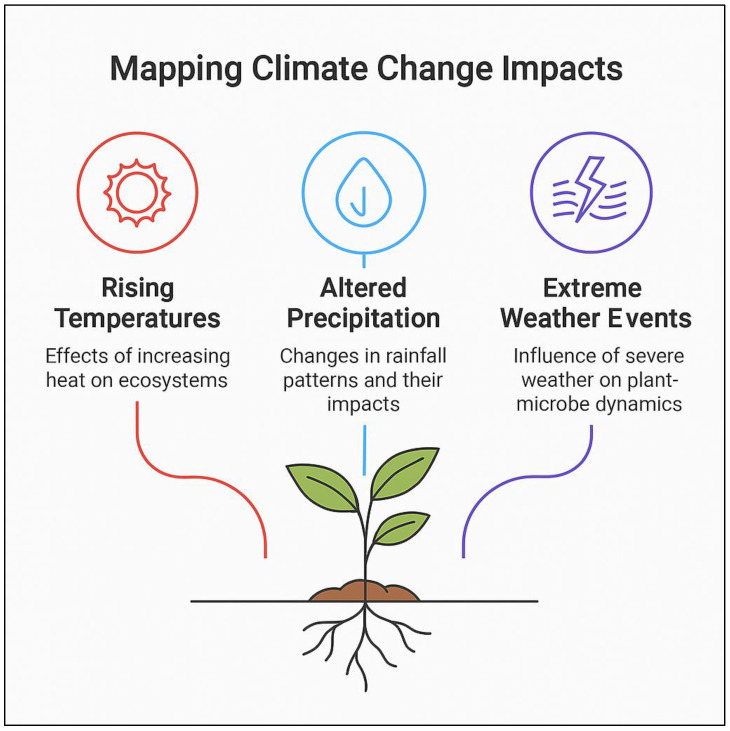
Mapping climate change impacts on plant–microbe interactions.

Driven by global warming, climate change, including altered precipitation patterns and the increased occurrence of extreme weather events, has profound implications for plant health and agricultural productivity. These changes not only affect plants directly by creating new stressors, such as extreme heat, drought, and flooding, but also have significant impacts on plant–microbe interactions [132,133]. The dynamic relationship between plants and their associated microbiomes is increasingly influenced by shifting climatic conditions, which alter the structure, function, and resilience of microbial communities. Understanding how climate change will affect these interactions is crucial for developing strategies to maintain or enhance plant productivity and ecosystem stability in our rapidly changing environment [134]. Figure 3 illustrates the various impacts of climate change on plant–microbe interactions, highlighting the effects of altered precipitation, rising temperatures, and extreme weather events on ecosystem dynamics.

Rising temperatures and altered precipitation patterns affect microbial communities in the soil and on plant surfaces, changing their abundance, diversity, and functionality. For instance, warmer temperatures may favor certain heat-tolerant microbial species, while others, particularly those sensitive to temperature extremes, may decline [135]. Similarly, shifts in precipitation can impact the distribution of microbes, with drought conditions potentially reducing microbial activity in the soil, while excessive rainfall could lead to waterlogging, affecting microbial community composition and nutrient cycling [136]. In the rhizosphere, drought can reduce the availability of water and nutrients, causing microbial populations to shift towards more drought-tolerant species, such as drought-resistant bacteria and fungi. At the same time, extreme weather events like floods or prolonged wet periods can encourage the growth of pathogenic microbes, altering the balance between beneficial and harmful microbes in the plant microbiome [137,138].

Furthermore, interactions between climate-induced stressors, such as elevated temperatures and increased salinity, can have synergistic effects on plant–microbe dynamics. For example, salinity stress, exacerbated by rising sea levels and reduced freshwater availability, can disrupt microbial symbioses by interfering with nutrient exchange and microbial colonization of plant roots. Jorquera et al. [139] demonstrated that increased salinity significantly alters rhizosphere microbial structure, reducing the abundance of beneficial microbes that promote plant growth [139]. Similarly, elevated atmospheric CO_2_ concentrations may influence plant–microbe interactions by modifying root exudation patterns, which in turn reshape microbial community composition and function. Goulden, et al. [140] showed that increased CO_2_ can enhance microbial activity but also reduce microbial functional diversity depending on plant species and soil type [140].

The impacts of climate change on plant–microbe interactions are highly dynamic, with shifts in microbial community structure and function directly influencing plant health, nutrient acquisition, and disease resistance. Zeng et al. [141] found that warming-induced shifts in soil microbiomes may decrease plant productivity by favoring microbes less involved in nutrient cycling [141]. As these interactions evolve under climate pressure, maintaining or enhancing the resilience of plant–microbe symbioses will be essential for sustaining crop production and biodiversity. Leveraging these interactions through targeted bioinoculants, climate-resilient microbial consortia, and microbiome engineering offers promising avenues to strengthen agricultural systems amid environmental change [142,143].

## 5. Advances in Microbiome Engineering for Stress Resilience

With the increasing recognition of the role plant-associated microbiomes play in stress mitigation, researchers have focused on developing innovative tools and technologies to manipulate and optimize microbial communities for improved plant performance [144]. These emerging tools—ranging from synthetic biology to advanced molecular profiling—are paving the way for more targeted and efficient approaches to stress resilience, with potential applications in both field-based agriculture and controlled environments [145]. Table 4 summarizes these tools and highlights their potential in enhancing plant stress tolerance.

### 5.1. Emerging Tools and Technologies

The development of cutting-edge technologies has allowed for the more precise and informed manipulation of plant microbiomes. Synthetic biology has become a cornerstone of microbiome engineering, enabling the design and construction of SynComs that can be tailored to meet the specific needs of plants under stress [154,155]. By combining multiple microbial species with complementary functions, SynComs can be engineered to optimize nutrient uptake, enhance stress tolerance, or promote plant growth under a range of environmental conditions [156]. Recent work by Schmitz, et al. [157] demonstrated the successful development and application of a synthetic microbial community derived from desert rhizosphere bacteria to improve tomato resilience under salinity stress. Their SynCom, composed of 15 selected strains, was applied to plants grown in non-sterile soil. Treated plants exhibited significantly increased shoot biomass and improved physiological traits under salt stress compared to untreated controls, indicating SynCom’s potential for stress mitigation through modulation of plant responses and nutrient acquisition [157]. Despite these promising results, the SynCom application still faces challenges. Field consistency remains difficult due to variable soil microbiomes, colonization efficiency, and microbial persistence. Future strategies must consider adaptive design, strain compatibility, and environmental context to ensure successful and reproducible SynCom performance in diverse agricultural systems. Synthetic biology also enables the incorporation of functional genes into microbial communities, allowing engineered microbes to perform specialized roles such as the degradation of harmful compounds or the production of protective metabolites. These engineered microbial communities have the potential to create more resilient crops by enhancing the natural symbiotic relationships between plants and microbes [158].

Another significant advancement is the application of metagenomics, transcriptomics, and metabolomics to microbiome profiling. These high-throughput technologies allow researchers to capture the full spectrum of microbial diversity, gene expression, and metabolic activity, respectively, of plant-associated microbiomes and microbes [159,160]. Metagenomics enables the sequencing of microbial DNA from environmental samples, providing a comprehensive view of the microbial community composition [161]; transcriptomics analyzes the gene expression patterns of microbes, revealing which genes are active under stress conditions; and metabolomics allows the identification of metabolites produced by both microbes and plants. These tools are invaluable for understanding molecular interactions between plants and their microbiomes, enabling the identification of key microbial species and metabolic pathways involved in stress resilience [162,163]. By combining these approaches, researchers can uncover new microbial functions that could be harnessed to improve plant performance under challenging environmental conditions.

### 5.2. Bioinoculants and Biostimulants for Field Applications

Bioinoculants and biostimulants are rapidly gaining attention as practical, sustainable solutions for enhancing plant growth and stress tolerance in agriculture. Bioinoculants consist of beneficial microorganisms, such as nitrogen-fixing bacteria, phosphate-solubilizing fungi, and PGPR, that are applied to soil or plant surfaces to enhance plant health [164]. These microbial inoculants improve nutrient availability, suppress pathogens, and help plants adapt to environmental stressors [165]. Microbes commonly used in bioinoculants include Rhizobium species, which form symbiotic relationships with leguminous plants to fix atmospheric nitrogen, and mycorrhizal fungi, which enhance phosphorus uptake and improve root architecture under drought conditions. In addition to improving nutrient acquisition, bioinoculants can enhance plant resistance to abiotic stresses, such as water scarcity, salinity, and temperature extremes [166,167].

**Figure 4:**
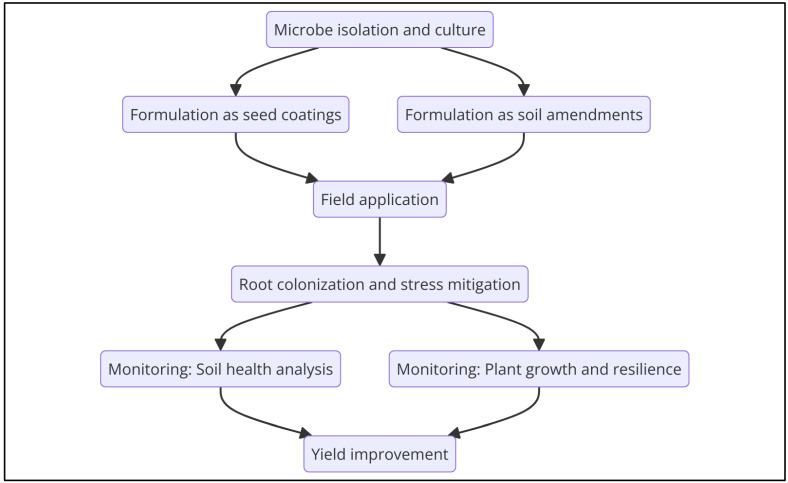
Comprehensive process of bioinoculant application for soil and crop enhancement.

Biostimulants, on the other hand, are products that contain living microorganisms or microbial metabolites designed to promote plant growth and stress tolerance. These products can include plant hormones, organic acids, and microbial exudates that stimulate plant immune responses, improve root growth, and enhance photosynthesis [168]. Their efficacy has been demonstrated in multiple field and greenhouse studies; for example, bacterial and fungal-based biostimulants have been shown to improve drought tolerance in wheat and salinity tolerance in tomato by modulating root architecture and ion transport pathways [169,170]. When applied in the field, biostimulants can enhance plant resilience to drought by improving root architecture and water uptake or mitigate the negative effects of salinity by promoting ion homeostasis. They offer a sustainable alternative to chemical fertilizers and pesticides, helping reduce the environmental impact of farming while improving crop yield and quality [171]. As the use of bioinoculants and biostimulants becomes more widespread, further research is needed to optimize their formulation, application, and efficacy under different environmental conditions [172]. Figure 4 provides a flowchart outlining a typical process for formulating and applying bioinoculants in agricultural settings.

### 5.3. CRISPR-Cas Tools for Microbial Enhancement

Exploring the complex interactions between plants and microorganisms requires advanced tools to uncover their critical genetic and molecular mechanisms. In this context, recently developed CRISPR/Cas-mediated genome editing technologies have gained significant attention for investigating and manipulating plant-microbe interactions. The CRISPR-Cas system can be used to edit microbial genomes for enhanced stress resilience. CRISPR-Cas has been successfully applied to modify the genomes of both plant and microbial species, enabling precise control over their traits [173]. In microbiome engineering, CRISPR-Cas can be used to insert, delete, or modify genes in microbial species to improve their stress tolerance, nutrient cycling capacity, or production of beneficial metabolites [174]. For example, microbial strains can be engineered to produce higher levels of osmoprotectants like trehalose during drought or synthesize phytohormones such as auxins to promote plant growth under nutrient-limited conditions [151]. Additionally, CRISPR-Cas can be used to enhance the ability of microorganisms to interact more effectively with plants, improving nutrient exchange and promoting plant immune responses. This level of precision opens up new possibilities for optimizing microbial communities to help plants cope with the stresses imposed by climate change [175,176].

The use of CRISPR-Cas systems also enables researchers to design synthetic microbial consortia with enhanced capabilities, such as increased tolerance to extreme temperatures or the ability to detoxify heavy metals in polluted soils. These microbial communities can be tailored to specific crops and environmental conditions, providing an efficient and scalable approach to improving plant resilience on a global scale [177]. While the application of CRISPR-Cas in microbiome engineering is still in its early stages, the potential for developing next-generation bioinoculants and biostimulants that incorporate CRISPR-edited microbes holds great promise for sustainable agriculture [178,179].

### 5.4. Crosstalk in Microbes and Climate Change Mitigation

Microbial interactions, including cooperation, competition, and synergism, can influence plant responses to environmental stressors [180]. For instance, microbial consortia in the rhizosphere can work together to enhance nutrient cycling, promote water retention, and suppress pathogenic microbes, all of which help plants cope with stress [181]. Microbial community structure and function are influenced by environmental factors such as temperature, moisture, and soil nutrients, which are all affected by climate change [182].

The interplay between plants and their associated microbial communities is also a crucial factor in climate change mitigation. Microbes can influence the plant’s ability to sequester carbon, reduce greenhouse gas emissions, and improve soil health [183]. For example, certain microorganisms play a role in the carbon cycle by enhancing soil carbon sequestration through improved organic matter decomposition and microbial mineralization [183]. Additionally, microbes involved in denitrification can reduce nitrogen oxide emissions, a potent greenhouse gas. By optimizing plant-microbe interactions through microbiome engineering, we can develop more resilient agricultural systems that contribute to mitigating climate change while enhancing food security [184,185].

## 6. Opportunities and Challenges in Utilizing Plant Microbiomes

**Figure 5:**
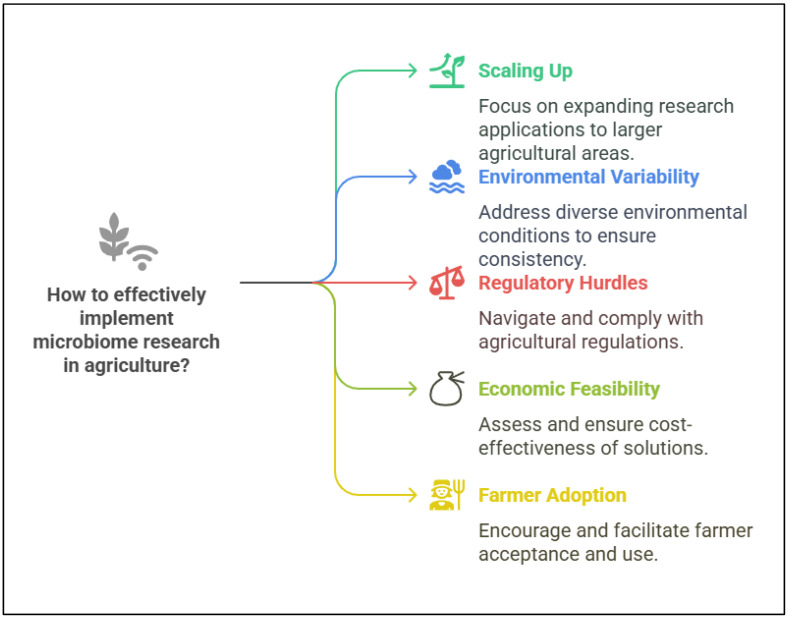
Key considerations for implementing plant microbiome research in agriculture.

The potential of plant microbiomes to enhance plant growth, increase stress resilience, and promote sustainable agricultural practices has garnered substantial attention in recent years [145]. However, while the understanding of microbiome functions in controlled laboratory settings has advanced significantly, translating these findings into practical, real-world agricultural applications remains a considerable challenge. Bridging the gap between lab-scale research and field-based implementation is essential for realizing the true potential of microbiomes in agriculture and climate change mitigation [186,187]. The effective utilization of plant microbiome research in agriculture requires addressing multiple challenges and opportunities (Figure 5). Key factors include scaling up research applications to larger agricultural systems, managing environmental variability to ensure consistency across diverse conditions, and navigating regulatory hurdles to comply with agricultural policies. Additionally, the economic feasibility of microbiome-based solutions must be evaluated to promote cost-effectiveness, while farmer adoption remains critical for the practical implementation and long-term sustainability of microbiome-based practices. Addressing these aspects holistically will be essential in leveraging plant microbiomes for sustainable agricultural advancements.

### Translating Lab-Scale Findings into Field Applications

Laboratory plant-microbiome research has provided valuable insights into how specific microbial communities can improve plant health, nutrient uptake, and stress resilience. In controlled environments, it is easier to identify microbial species that enhance plant performance by controlling environmental factors such as temperature, soil composition, moisture, and nutrient availability [188]. However, in field conditions, these factors are highly variable and unpredictable, complicating the ability to achieve consistent results with microbiome interventions. For instance, beneficial microbes that thrive under lab conditions may struggle in the diverse and dynamic environments of field soils, where native microbial populations and environmental factors like soil pH, organic matter, and moisture content play a significant role [189,190].

Moreover, the efficacy of microbial inoculants is influenced by factors such as soil variability, climate conditions, and soil health, and field conditions can differ greatly from those under which laboratory trials are conducted. Soil heterogeneity and environmental stressors, such as extreme heat, drought, or flooding, can influence microbial survival and activity, making it difficult to predict how microbial solutions will perform across different regions, climates, and crop types [191,192]. Therefore, a key challenge in scaling up microbiome interventions is ensuring that they are robust enough to handle the complex, variable conditions encountered in the field. Achieving consistent microbial performance will require further research on soil–microbe interactions, environmental adaptability, and the long-term persistence of microbial strains under field conditions [193].

Another challenge lies in optimizing application methods for microbiome-based solutions. Field trials often face practical difficulties related to the delivery, incorporation, and maintenance of microbial inoculants [194]. Unlike laboratory settings where microorganisms can be carefully applied and monitored, field applications must address issues such as inoculant survivability, compatibility with farming practices, and economic feasibility. This necessitates the development of more scalable and efficient delivery methods, such as seed coatings, soil amendments, or foliar sprays, that can ensure successful establishment in the field [195,196].

Despite these obstacles, the integration of microbial-based solutions into agricultural systems holds immense promise, and overcoming barriers to field application can lead to significant advances in sustainable agriculture [156]. Researchers are now focused on developing better, more field-adapted bioinoculants and biostimulants with enhanced microbial resilience to environmental stressors [197]. By leveraging microbial diversity and synthetic biology, future innovations in microbiome engineering can offer customized solutions tailored to specific crop species, regions, and stress conditions [156].

## 7. Final Remarks and Future Perspectives

### 7.1. Reiterating the Importance of Plant Microbiomes in Building Climate-Resilient Systems

Plant microbiomes play vital roles in nutrient cycling, stress mitigation, and disease resistance. Plant-microbiome interactions help maintain soil health, enhance water and nutrient uptake, and protect against environmental stressors such as drought, salinity, and extreme temperatures [134]. As climate change intensifies and places additional pressures on agriculture, microbiomes offer a natural and sustainable approach to building climate-resilient agricultural systems. Harnessing their power can help farmers adapt to changing environmental conditions, improve crop yield stability, and reduce the need for chemical inputs [198,199].

### 7.2. Call to Action for Interdisciplinary Research and Collaboration

The potential of plant microbiomes to contribute to sustainable agriculture and environmental resilience underscores the importance of interdisciplinary collaboration. Advances in microbiome science require collaboration across fields, including plant biology, microbiology, ecology, and environmental science [156,200]. Researchers, policy-makers, and agricultural stakeholders must work together, sharing knowledge, resources, and technologies that can drive the integration of microbiome-based solutions into farming practices [201]. As our understanding of microbiomes evolves, it will be crucial to foster collaborative partnerships between academia, industry, and government to develop innovative, scalable solutions that can be applied across diverse agricultural systems.

One key area for interdisciplinary collaboration is the development of regulatory frameworks for the safe and effective use of microbial products in agriculture. As microbiome-based solutions move from the laboratory to the field, it is essential to ensure their safety and efficacy through rigorous testing and regulatory oversight [202]. Collaboration with regulatory bodies can help standardize protocols for the production, application, and monitoring of microbial inoculants and biostimulants, ensuring that these solutions meet the necessary health and environmental standards while also providing measurable benefits to crops [203,204].

### 7.3. Role of Plant Microbiomes in Sustainable Agriculture and Ecosystem Restoration

Microbial communities are at the forefront of sustainable agriculture and ecosystem restoration. By promoting soil health, enhancing nutrient cycling, and reducing the need for chemical fertilizers, plant microbiomes offer a safe and sustainable alternative to conventional farming practices [205]. The role of microbiomes in soil carbon sequestration, nitrogen fixation, and pathogen suppression can contribute significantly to reducing the environmental footprint of agriculture [206]. Notably, the Fabaceae family (legumes) plays a vital role in sustainable agriculture due to its ability to establish symbiotic nitrogen-fixing relationships with rhizobia, including genera such as *Rhizobium*, *Bradyrhizobium*, *Mesorhizobium*, and *Sinorhizobium*. These bacteria colonize specialized root structures called nodules, where they convert atmospheric nitrogen into forms usable by plants [100]. This natural fertilization process reduces dependency on synthetic nitrogen fertilizers, enhances soil fertility, and supports crop rotations that improve overall agroecosystem health. In addition, microbiomes can also aid in ecosystem restoration by improving soil fertility and promoting plant growth in degraded or polluted soils. Microbial inoculants can be used in reforestation projects, land reclamation, and the restoration of ecosystems affected by mining or industrial activity, improving soil quality and supporting the re-establishment of native vegetation [205].

### 7.4. Integrating Microbiome Research with Climate-Smart Agricultural Practices

To fully harness the agricultural potential of microbiomes, it is essential to integrate microbiome research with climate-smart agricultural practices. Climate-smart agriculture focuses on increasing agricultural productivity, enhancing resilience to climate change, and reducing greenhouse gas emissions [207,208]. By incorporating microbiome-based solutions into climate-smart agriculture, we can improve resource use efficiency, increase plant resistance to environmental stress, and reduce the need for harmful chemical inputs [209]. For example, microbial biostimulants and bioinoculants can improve water-use efficiency in drought-prone areas, while nitrogen-fixing bacteria can reduce the need for synthetic fertilizers in nutrient-deficient soils. The integration of microbiomes into sustainable farming practices can thus help meet global food security goals while mitigating the environmental impact of agriculture [210,211].

### 7.5. Policy and Funding Priorities for Microbiome-Based Solutions

To fully realize the potential of plant microbiomes, policy and funding priorities must align with the goal of advancing microbiome-based solutions in agriculture. Governments and funding agencies should prioritize research that addresses key knowledge gaps in microbiome science, such as the lack of knowledge regarding the long-term effects of microbiome-based interventions on plant health and soil ecosystems [203,212]. Investment in biotech innovation and the development of scalable microbiome-based products is essential to the commercialization of these solutions [202]. Additionally, policy frameworks should encourage the adoption of sustainable agricultural practices that leverage microbiomes, including incentives for farmers to incorporate bioinoculants and biostimulants into their crop management strategies. Public-private partnerships can play a vital role in fostering innovation and bringing microbiome-based solutions to market, ensuring that these technologies are accessible, affordable, and effective for farmers worldwide [156,213,214].

## 8. Concluding Remarks

It is becoming more obvious how plant microbiomes contribute to improved plant health, stress tolerance, and overall agricultural sustainability. Experimental studies have shown that plant-associated microbial communities, especially those in the rhizosphere and endosphere, contribute significantly to nutrient cycling, hormonal regulation, antioxidant activation, and stress recovery in crops under drought, salinity, and extreme temperatures. These microbiome-mediated effects have been demonstrated in crops like rice, wheat, and maize, where specific microbial consortia promote root development, improve water-use efficiency, and enhance plant tolerance to oxidative damage. However, challenges remain in transferring lab-scale findings to field conditions due to variability in soil, climate, and host-microbiome compatibility. Recent tools such as metagenomics, synthetic microbial consortia, and field-scale microbiome profiling are beginning to bridge these gaps. To ensure practical impact, microbiome-based approaches must be evaluated under diverse environmental settings and supported by robust experimental validation and interdisciplinary research efforts. With global food systems increasingly affected by climate change, leveraging plant microbiomes offers a science-driven, sustainable pathway forward. Achieving this potential will require coordinated action from researchers, policymakers, and farmers to implement microbiome-informed agricultural strategies that are scalable, reproducible, and climate-resilient.

## Figures and Tables

**Table 1:** Overview of major abiotic stresses and their primary physiological impacts on plants.

Abiotic Stress	Physiological Impact	References
Drought	Reduced water availability, stomatal closure, decreased photosynthesis, oxidative stress, and leaf wilting.	[38]
Salinity	Osmotic stress, ionic imbalance (Na+ and Cl− toxicity), reduced water uptake, impaired photosynthesis.	[39]
Extreme Heat	Protein denaturation, membrane fluidity disruption, increased transpiration, oxidative damage.	[40]
Extreme Cold	Membrane rigidification, reduced enzymatic activities, decreased photosynthesis.	[41]
Nutrient Deficiency	Limited chlorophyll production, impaired metabolic pathways, reduced growth and yield.	[42]
Waterlogging	Oxygen deprivation in roots, reduced nutrient uptake, increased ethylene production.	[43]

**Table 2:** Key microbial mechanisms for alleviating abiotic stress in plants.

Microbial Mechanism	Description	References
Enhancing Nutrient Uptake	Microbes like mycorrhizal fungi and rhizobacteria improve nutrient availability (e.g., phosphorus and nitrogen) and facilitate uptake.	[8]
Phytohormone Modulation	Microbial production of auxins, gibberellins, and cytokinins regulates plant growth, while ACC deaminase-producing bacteria reduce ethylene stress.	[92]
Osmoprotectant Production	Bacteria produce osmolytes (proline, trehalose) to help plants maintain water balance under drought and salinity stress.	[93]
Inducing Systemic Tolerance (IST)	Rhizobacteria trigger plant defense responses, improving resistance to drought, salinity, and heat stress.	[94]
Exopolysaccharide (EPS) Production	Microbial EPS helps in water retention around plant roots, preventing desiccation under drought conditions.	[95]
Antioxidant Enzyme Activation	Microbes enhance the activity of superoxide dismutase (SOD) and catalase (CAT), reducing oxidative stress.	[96]
Heavy Metal Detoxification	Certain microbes sequester toxic heavy metals through biosorption, enhancing plant survival in contaminated soils.	[97]

**Table 3:** Examples of microbial strategies for alleviating heavy metal stress.

Microbial Strategy	Description	Examples	References
Biosorption	Bacteria and fungi absorb and immobilize heavy metals through their cell walls, reducing metal toxicity in plants.	*Pseudomonas putida* efficiently removes cadmium (Cd) from contaminated soils.	[124]
Bioaccumulation	Microbes internalize heavy metals, preventing uptake by plants.	*Bacillus subtilis* accumulates arsenic (As), reducing its availability in rice fields.	[125]
Biotransformation	Enzymatic conversion of toxic metals into less harmful forms (e.g., reduction of Cr^6+^ to Cr^3+^).	*Pseudomonas aeruginosa* converts toxic Cr^6+^ to Cr^3+^, reducing its toxicity.	[126]
Exopolysaccharide (EPS) Production	Microbial EPS binds heavy metals, preventing their transport into plant tissues.	*Azotobacter chroococcum* produces EPS that binds Pb, Zn, and Cd, reducing plant uptake.	[127]
Metal Precipitation	Sulfate-reducing bacteria precipitate heavy metals as insoluble sulfides, limiting bioavailability.	*Desulfovibrio desulfuricans* reduces U^6+^ to insoluble U^4+^ in uranium-contaminated soils.	[128]
Rhizoremediation	Rhizobacteria enhance metal uptake and promote plant growth.	*Rhizobium leguminosarum* assists pea plants in lead (Pb) tolerance and uptake reduction.	[129]
Chelation & Siderophore Production	Microbial siderophores chelate heavy metals, reducing toxicity and promoting sequestration.	*Pseudomonas fluorescens* produces siderophores that bind iron (Fe) and lead (Pb).	[130]
Genetically Engineered Microbes	Engineered microbes enhance metal tolerance and detoxification in contaminated soils.	*Escherichia coli* engineered to express metallothioneins for cadmium (Cd) detoxification.	[131]

**Table 4:** Representative tools and technologies for microbiome engineering.

Tool/Technology	Description	Potential Application	References
Metagenomics	High-throughput sequencing to profile entire microbial communities in plant-associated environments.	Identifies beneficial microbes that enhance drought and salt stress tolerance.	[146]
Transcriptomics	RNA sequencing to analyze microbial gene expression under different stress conditions.	Determines microbial responses to environmental stressors, guiding microbiome engineering.	[147]
Metabolomics	Analyzes plant-microbe metabolite interactions to understand stress adaptation.	Identifies microbial metabolites that promote stress tolerance in plants.	[148]
Proteomics	Large-scale study of microbial and plant protein expression under stress conditions.	Identifies functional proteins and stress-responsive pathways involved in microbiome-mediated plant resilience.	[149]
Synthetic Microbial Communities (SynComs)	Assembles beneficial microbial consortia to improve plant resilience.	Enhances crop productivity by introducing beneficial microbiomes in degraded soils.	[150]
CRISPR-based Microbiome Editing	Uses CRISPR-Cas systems to engineer beneficial microbial strains for stress resistance.	Enhances microbial traits that help plants tolerate extreme environmental conditions.	[151]
Artificial Microbial Consortia	Designs microbial communities with specific plant-growth-promoting traits.	Introduces engineered microbes that increase plant stress tolerance.	[152]
Bioinformatics & Machine Learning	Uses computational models to predict plant-microbiome interactions.	Optimizes microbiome engineering strategies for targeted plant benefits.	[153]
